# Development and validation of a predictive model for forceps delivery risk in term singleton primiparas for early identification and labor management optimization

**DOI:** 10.3389/fmed.2026.1840918

**Published:** 2026-06-19

**Authors:** Fengchao Wan, Xiaoqin Chen, Xiaojing Song, Xue Mei, Qi Shi

**Affiliations:** 1 Department of Obstetrics, The Second People’s Hospital of Yibin, Yibin, China; 2 College of Pharmacy, North Sichuan Medical College, Nanchong, China; 3 Department of Obstetrics, Affiliated Hospital of North Sichuan Medical College, Nanchong, China

**Keywords:** forceps delivery, nomogram, prediction model, primipara, risk factors

## Abstract

**Introduction:**

This study aimed to establish and validate a predictive model for forceps-assisted delivery in term singleton primiparas during the early second stage of labor, enabling early identification of high-risk cases and targeted intrapartum care to reduce maternal and neonatal risks associated with prolonged labor.

**Materials and methods:**

The study retrospectively analyzed clinical data from pregnant women who delivered at The Second People’s Hospital of Yibin City between January 2017 and December 2024. Term singleton primiparas who underwent forceps delivery were designated as the forceps delivery group (*n* = 346), while each corresponding case of spontaneous vaginal delivery (SVD) was included in the vaginal delivery group (*n* = 346) using adjacent case matching. Maternal, fetal, and labor characteristics were collected and compared between groups. Univariate and multivariate logistic regression analyses were conducted to identify independent risk factors for forceps delivery and to construct a predictive model. Model performance was comprehensively evaluated in terms of discrimination, calibration, and clinical utility using the area under the receiver operating characteristic curve (AUC), the Hosmer–Lemeshow test, the Brier score, and clinical decision curve analysis (DCA).

**Results:**

Independent risk factors for forceps delivery in term singleton primiparas identified at the onset of the second stage of labor included: pre-delivery body mass index (BMI) ≥ 28 kg/m^2^, fetal abdominal circumference ≥ 340 mm, grade II or III meconium-stained fluid (MSF), non-occipital anterior (OA) fetal position, and active phase duration ≥ 120 min. This predictive model for early risk identification during the second stage of labor achieved an AUC of 0.775 (95% CI: 0.741–0.810), with an optimal cutoff value of 0.413, corresponding to a sensitivity of 0.688 and a specificity of 0.725. The Hosmer–Lemeshow test indicated a good model fit. After 500 internal bootstrap validations, the corrected C-index was 0.771. The model demonstrated acceptable calibration and provided a net benefit over the treat-all strategy for clinically plausible threshold probabilities up to 0.35.

**Conclusion:**

The proposed model, based on routine intrapartum predictors at the onset of the second stage, enables early risk stratification for forceps delivery in term singleton primiparas and supports anticipatory intrapartum planning. Further prospective, multicenter external validation is warranted to determine its real-world clinical impact and generalizability.

## 1 Introduction

In recent years, assisted vaginal birth rates have been declining globally, while cesarean delivery rates continue to rise (1). This trend has prompted a renewed focus on reducing cesarean section rates and promoting vaginal birth in contemporary obstetric care. Timely and standardized assisted vaginal delivery is critical for lowering the primary cesarean rate and efficiently managing second-stage labor dystocia. In appropriately selected cases and when performed by experienced operators under standardized protocols, forceps-assisted delivery may shorten the second stage of labor and help prevent emergency cesarean delivery (2–4).

However, the maternal and neonatal benefits and risks of forceps-assisted delivery, vacuum extraction, and cesarean delivery are context-dependent and may vary according to indication, fetal station and fetal position, operator experience, and institutional practice. Current clinical decision-making regarding forceps-assisted delivery largely relies on obstetricians’ subjective experience, and objective, quantifiable risk assessment tools remain limited. Although several studies have identified risk factors associated with forceps-assisted delivery, few have established predictive models that can be practically applied at the onset of the second stage of labor (5, 6). This gap may lead to insufficient preparation or delayed mobilization of experienced obstetric and neonatal support teams, potentially affecting the timeliness and safety of intrapartum management.

Therefore, to enable early identification and precise management of risks associated with forceps-assisted delivery, this study retrospectively analyzed clinical data from women who underwent forceps-assisted and spontaneous vaginal deliveries, focusing on the critical decision-making point at the onset of the second stage of labor. The study aimed to identify independent predictors of forceps-assisted delivery and develop an interpretable nomogram model for early intrapartum risk stratification. This model aims to help the midwifery team identify high-risk mothers in advance, enabling the timely mobilization of qualified forceps operators and neonatal resuscitation teams. This approach may help optimize intrapartum resource preparation, although its impact on maternal and neonatal outcomes needs prospective implementation studies.

## 2 Materials and methods

### 2.1 Study participants and grouping

From January 2017 to December 2024, clinical data obtained from pregnant women who delivered at The Second People’s Hospital of Yibin City were retrospectively collected. The study included term singleton primiparas who underwent forceps delivery (*n* = 346) as the forceps delivery group. For each index case in the forceps group, the next eligible pregnant woman who achieved spontaneous vaginal delivery (SVD) without instrumental assistance was selected using adjacent case matching to balance confounding related to delivery time and intrapartum management between the two groups, forming the SVD group (*n* = 346), thereby minimizing temporal variations in obstetric practice and staffing. The two groups were matched by delivery time and managed within the same clinical setting. Adjacent case matching was used only as a sampling strategy to reduce temporal variation in obstetric practice and staffing. It was not intended to estimate the real-world prevalence of forceps-assisted delivery or to create a paired analytic structure.

The inclusion criteria were as follows: (1) full-term primipara; (2) cephalic presentation, singleton, live-born infant; (3) the forceps delivery group consisted of women with clear indications for assisted vaginal delivery (e.g., fetal distress or prolonged second stage of labor), with the indication and decision confirmed by a senior obstetrician experienced in operative vaginal delivery; (4) the spontaneous delivery group included women with normal fetal heart rate (FHR) patterns and labor progression, who delivered without instrumental assistance and received standard intrapartum care; and (5) complete delivery data were available for analysis.

The exclusion criteria were as follows: (1) vaginal birth after cesarean; (2) cervical dilation > 6 cm at admission; (3) induction of labor due to fetal malformation; and (4) SVD following the onset of indications for forceps assistance during labor.

### 2.2 Research methods and data collection

Patient demographics: maternal age, gestational age at delivery, pre-delivery BMI, obstetric ultrasound findings within 1 week prior to admission (including fetal abdominal circumference, amniotic fluid volume, and nuchal cord), and antepartum comorbidities were documented.

Delivery information: intrapartum interventions (including induction of labor and use of analgesia), obstetric conditions (e.g., premature rupture of membranes), fetal status at the onset of the second stage of labor (station, fetal position, and amniotic fluid status), as well as labor duration and neonatal birth weight, were also recorded.

### 2.3 Definition of variables

(1) Prolonged second stage of labor: primiparous using labor analgesia for more than 4 h in the second stage of labor, or those not using labor analgesia for more than 3 h (7);
(2) Fetal distress: diagnosed by the obstetrician based on abnormal FHR monitoring during labor (e.g., recurrent variable decelerations or frequent late decelerations) (8);
(3) Pre-delivery BMI: the ratio of the weight before delivery (kg) to the square of the height (m^2^) (9);
(4) MSF: Grade II (green- or yellow-stained amniotic fluid with some particulate matter); and grade III (brownish-yellow amniotic fluid with dense meconium) (10);
(5) Active phase duration: the duration from 6 cm cervical dilation to the start of the second stage of labor;
(6) Labor analgesia: patient-controlled epidural analgesia (PCEA) initiated when the cervix is dilated to 2–3 cm;
(7) Non-occiput anterior positions (non-OA): the fetal occipital region is located in front of the maternal pelvis for OA. Non-OA positions include occipital transverse and occipital posterior positions (11)^;^
(8) Fetal abdominal circumference ≥340 mm: measured by ultrasound within 1 week prior to admission. This variable is treated as a predictive indicator of relatively large fetal size at the onset of the second stage of labor and is distinct from both antenatal suspected macrosomia and postnatal macrosomia. It was included as a candidate predictor in the multivariable model;
(9) Postnatal macrosomia (12): newborn birth weight ≥ 4,000 g. Because this variable was available only after delivery, it was used solely for descriptive comparison between groups and was not included as a candidate predictor in the model developed for use at the onset of the second stage of labor;
(10) Oligohydramnios: ultrasound-measured amniotic fluid index ≤ 5 cm.

### 2.4 Statistical analysis

The results were analyzed using SPSS 25.0 and R 4.3.2. Normally distributed continuous variables were presented as mean ± standard deviation (SD) and compared between groups using the independent samples *t*-test. Non-normally distributed continuous variables were analyzed using the Mann–Whitney *U*-test and presented as median and interquartile range [M (Q1, Q3)]. The count data were described using frequencies and percentages. Chi-square (*χ*^2^) tests were used for intergroup comparisons.

For clinical interpretability and bedside applicability, selected continuous variables were dichotomized before model construction. Maternal age was dichotomized at 35 years based on the commonly used definition of advanced maternal age, and gestational age was dichotomized at 280 days, corresponding to 40 weeks of gestation. Pre-delivery BMI was dichotomized at 28 kg/m^2^ according to the obesity threshold used in the Chinese population. Fetal abdominal circumference and active phase duration were dichotomized at 340 mm and 120 min, respectively, based on the distribution of the study data and their clinical practicality for intrapartum risk assessment.

Variables with a *p*-value of < 0.05 in the univariate analysis were included in a multivariate logistic regression analysis using the forward stepwise method to screen for independent risk factors for forceps-assisted delivery in primiparas and to construct a predictive model. Collinearity diagnostics were performed for all candidate variables, with a variance inflation factor (VIF) < 5 considered indicative of the absence of severe collinearity; variables meeting this criterion were retained for the identification of independent risk factors.

The model’s performance was assessed by plotting the receiver operating characteristic (ROC) curve and evaluating its discrimination ability using the area under the curve (AUC). The Hosmer–Lemeshow test, calibration curve, Brier score, calibration intercept, and calibration slope were used to comprehensively evaluate model calibration. The nomogram prediction model was constructed using R 4.3.2, and its predictive performance was evaluated using the Concordance index (C-index). The optimal cutoff value of the model was determined using the Youden index (sensitivity + specificity-1). Internal validation was performed using bootstrap resampling (500 iterations) to assess the model’s stability and accuracy. A *p*-value of < 0.05 was considered statistically significant. The clinical net benefit of the predictive model was evaluated using decision curve analysis (DCA). Because the 1:1 adjacent case-matched sampling strategy artificially altered the prevalence of forceps-assisted delivery, DCA was interpreted cautiously and focused on clinically plausible threshold probabilities rather than the full mathematically positive range of net benefit.

## 3 Results

### 3.1 Univariate analysis of continuous variables

Neonatal birth weight, which was normally distributed, was compared using the independent samples *t*-test. In contrast, other variables with non-normal distributions were analyzed using the Mann–Whitney *U*-test. Maternal age at delivery, pre-delivery BMI, gestational age at delivery, fetal abdominal circumference, and active phase duration differed significantly between groups (*p* < 0.05, as shown in Table 1).

**Table 1:** Univariate analysis of continuous variables [M(QL, QU), $\bar{x}$ ± s].

Influencing factors	Vaginal delivery group	Forceps-assisted delivery group	*z*/*t*	*p*
Maternal age (years old)	26.000 (24.0, 29.0)	27.000 (24.0, 29.0)	−2.383	0.017
Pre-delivery BMI (kg/m^2^)	25.185 (23.4, 27.0)	26.346 (24.4, 28.4)	−5.331	0.000
Gestational age (d)	274.000 (269.0, 279.0)	276.000 (270.0, 281.0)	−2.531	0.011
Fetal abdominal circumference (mm)	333.000 (326.0, 338.0)	336.000 (329.0, 345.0)	−5.695	0.000
Active phase duration (min)	75.000 (55.0, 110.0)	110.000 (60.0, 170.0)	−5.190	0.000
Neonatal birth weight (g)	3,114.49 ± 313.99	3,228.45 ± 389.18	−4.239	0.000

### 3.2 Univariate analysis of count data

The core positioning of this study is to serve as a bedside rapid assessment tool for the risk of forceps-assisted delivery at the onset of the second stage of labor. To prioritize clinical operability in intrapartum emergency scenarios, ensure stable and reproducible model results, and enhance the feasibility of clinical translation, the study deliberately chose to construct the model using only binary variables. Continuous variables with a *p*-value of < 0.05 in the univariate analysis were converted into binary variables. Age was stratified at 35 years (advanced maternal age), BMI at 28 kg/m^2^ (obesity), and gestational age at 280 days (expected date of confinement). Neonatal birth weight was categorized as macrosomia. Fetal abdominal circumference and active phase duration were set as cutoff values based on the median values of the forceps-assisted delivery group: 340 mm and 120 min, respectively. The results showed that: advanced maternal age, pre-delivery BMI ≥ 28 kg/m^2^, gestational age ≥ 280 days, fetal abdominal circumference ≥ 340 mm, gestational diabetes mellitus (GDM), postnatal macrosomia, grade II or III MSF at the onset of the second stage of labor, non-OA fetal position at the onset of the second stage of labor, station ≤ 1 at the onset of the second stage of labor, and active phase duration ≥ 120 min were statistically significant (*p* < 0.05).

The following factors were not statistically significant (*p* > 0.05): premature rupture of membranes, intrahepatic cholestasis of pregnancy (ICP), induction of labor with uterotonics, labor analgesia, gestational hypertension, hypothyroidism in pregnancy, oligohydramnios, and a nuchal cord entanglement by two turns (Table 2).

**Table 2:** Univariate analysis of count data [M(QL, QU)].

Influencing factors	Vaginal delivery group (*n* = 346)	Forceps-assisted delivery group (*n* = 346)	*χ* ^2^	*p*
Advanced-maternal-age	4 (1.16)	13 (3.76)	4.885	0.027
Pre-delivery BMI ≥ 28 kg/m^2^	51 (14.74)	108 (31.21)	26.530	0.000
Gestational age ≥ 280 d	85 (24.57)	121 (34.97)	8.958	0.003
Fetal abdominal circumference ≥ 340 mm	62 (17.92)	145 (41.91)	47.484	0.000
Gestational diabetes mellitus (GDM)	61 (17.63)	84 (24.28)	4.615	0.032
Premature rupture of membranes (PROM)	80 (23.12)	95 (27.46)	1.721	0.190
ICP	24 (6.94)	29 (8.38)	0.511	0.475
Gestational hypertension	13 (3.76)	10 (2.89)	0.405	0.525
Hypothyroidism in pregnancy	89 (25.72)	76 (21.97)	1.345	0.246
Oligohydramnios	18 (5.20)	29 (8.38)	2.762	0.097
Labor induction	65 (18.79)	79 (22.83)	1.719	0.190
Labor analgesia	261 (75.43)	262 (75.72)	0.008	0.929
Nuchal cord entanglement by two turns	9 (2.60)	16 (4.62)	2.033	0.154
Postnatal macrosomia	1 (0.29)	8 (2.31)	5.516	0.019
MSF of Grade II or III	25 (7.23)	91 (26.30)	45.114	0.000
Non-OA fetal position	9 (2.60)	82 (23.70)	67.427	0.000
Station ≤ 1	133 (38.44)	201 (58.09)	26.761	0.000
Active phase duration ≥120 min	82 (23.70)	165 (47.69)	43.372	0.000

### 3.3 Multivariate analysis and establishment of a prediction model

#### 3.3.1 Multivariate analysis

Variables with a *p*-value of < 0.05 in the univariate analysis were included for further analysis. Collinearity diagnostics showed that the VIF for all variables was less than 5, indicating no multicollinearity (Table 3). Forward stepwise logistic regression was applied to identify independent risk factors, and five independent risk factors (*p* < 0.05) were ultimately identified: pre-delivery BMI ≥ 28 kg/m^2^, fetal abdominal circumference ≥ 340 mm, grade II or III meconium-stained amniotic fluid at the onset of the second stage of labor, non-occipitoanterior (non-OA) fetal position at the onset of the second stage of labor, and active phase duration ≥ 120 min. Variables that were statistically significant in the univariate analysis but excluded from the final model showed a *p*-value of > 0.05 in the multivariate regression and had no independent predictive value. These five independent risk factors were used as the independent variables, and forceps-assisted delivery in primiparas was used as the dependent variable. The assignment of each variable is shown in Table 4.

**Table 3:** Collinearity diagnosis for candidate predictors.

Influencing factors	VIF	Tolerance
Maternal age ≥35 years	1.035	0.966
Pre-delivery BMI ≥ 28 kg/m^2^	1.078	0.928
Gestational age ≥280 d	1.087	0.920
GDM	1.067	0.937
Fetal abdominal circumference ≥340 mm	1.164	0.859
MSF grade II or III	1.040	0.962
Non-OA fetal position	1.203	0.832
Station ≤1	1.168	0.856
Active phase duration ≥120 min	1.163	0.859

**Table 4:** Variable assignments for the final multivariable logistic regression model.

Influencing factors	Variables	Assignment
Forceps-assisted delivery	Y	0 = no, 1 = yes
Pre-delivery BMI ≥ 28 kg/m^2^	X1	0 = no, 1 = yes
Fetal abdominal circumference ≥ 340 mm	X2	0 = no, 1 = yes
MSF of Grade II or III	X3	0 = no, 1 = yes
Non-OA fetal position	X4	0 = no, 1 = yes
Active phase duration ≥ 120 min	X5	0 = no, 1 = yes

Predictive model for forceps-assisted delivery in vaginal births of primipara (Table 5). The logistic regression model is: $Y=\frac{\exp\;(x)}{1+\exp\;(x)}$ , *X* = −1.189 + 1.030 × *X*1 + 1.012 × *X*2 + 1.481 × *X*3 + 2.048 × *X*4 + 0.711 × *X*5.

**Table 5:** Multivariate logistic regression analysis.

Influencing factors	*β*	S.E.	Wald	*p*	OR	95% CI
Pre-delivery BMI ≥ 28 kg/m^2^	1.030	0.213	23.331	0.000	2.802	1.845–4.257
Fetal abdominal circumference ≥ 340 mm	1.012	0.197	26.313	0.000	2.750	1.868–4.048
MSF of grade II or III	1.481	0.262	32.019	0.000	4.397	2.632–7.343
Non-OA fetal position	2.048	0.379	29.170	0.000	7.750	3.686–16.294
Active phase duration ≥ 120 min	0.711	0.190	13.965	0.000	2.036	1.402–2.956
Constant	−1.189	0.136	76.768	0.000	0.305	

#### 3.3.2 The establishment of a risk nomogram prediction model

**Figure 1:**
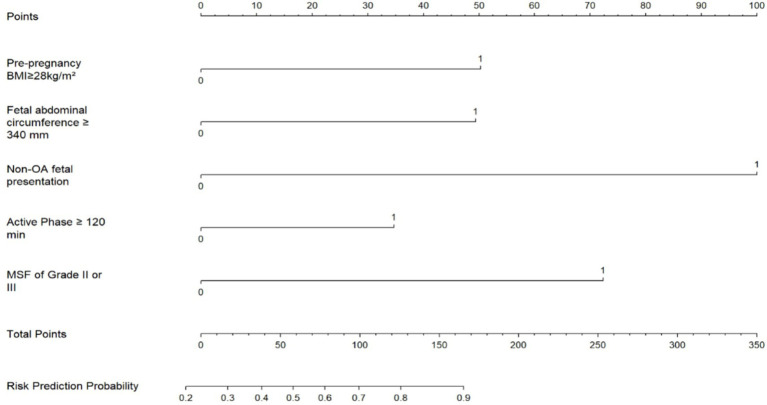
Nomogram for predicting forceps delivery in term singleton primiparas at the onset of the second stage of labor. The nomogram was constructed based on five independent predictors identified by multivariable logistic regression: pre-delivery BMI ≥ 28 kg/m^2^, fetal abdominal circumference ≥340 mm, grade II or III MSF, non-occiput anterior (non-OA) fetal position, and active phase duration ≥120 min. The total points correspond to the estimated individual probability of forceps delivery.

Utilizing R 4.3.2 software and the rms package, a risk nomogram prediction model for forceps-assisted delivery in primipara was constructed (Figure 1). This prediction model transforms each risk factor into an intuitive score. By accumulating the corresponding points assigned on the score axis, a total score was obtained. The risk value corresponding to this total score on the risk axis yields the predicted probability. The nomogram is presented in a visual format, aiding obstetricians in quickly and intuitively assessing the impact of different risk factors on forceps-assisted delivery, thereby enhancing the efficiency of clinical decision-making.

### 3.4 Prediction model evaluation

#### 3.4.1 Prediction performance evaluation

**Figure 2:**
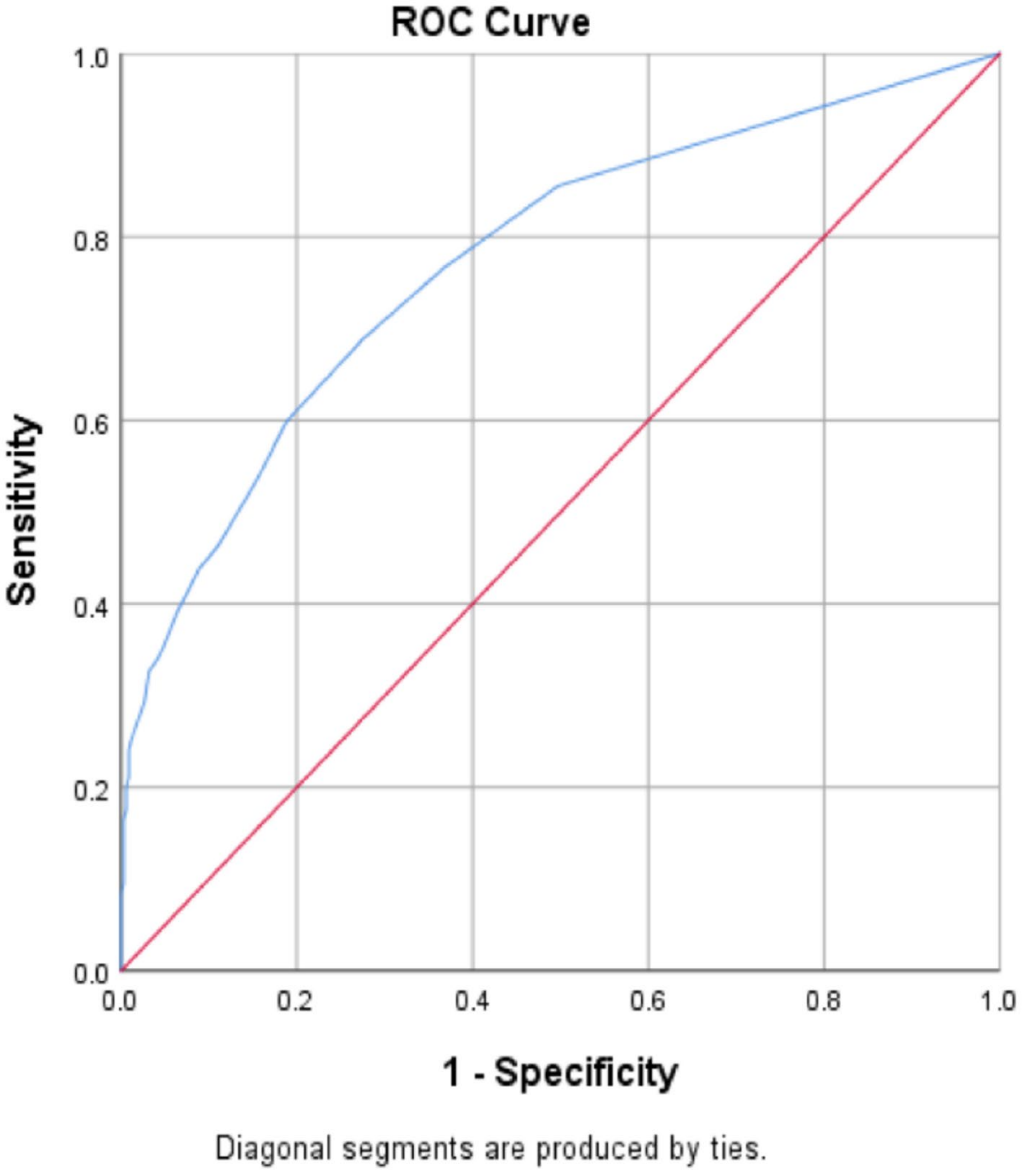
ROC curve of the prediction model for forceps-assisted delivery. The model showed good discriminatory ability, with an AUC of 0.775 (95% CI, 0.741–0.810). The optimal cutoff value was 0.413, yielding a sensitivity of 0.688 and a specificity of 0.725.

Using the calculated results of the prediction model as the test variable and whether forceps-assisted delivery was performed as the state variable, an ROC curve was constructed (Figure 2). The AUC was 0.775 (95% Cl: 0.741–0.810), indicating that the model has good predictive performance. The corresponding cutoff value was 0.413, with a sensitivity of 0.688 and a specificity of 0.725. The Omnibus test yielded *χ*^2^ = 188.496, *p* = 0.000, indicating that the model construction is statistically significant. The Hosmer–Lemeshow test result was *χ*^2^ = 3.214, *p* = 0.782, confirming that the model’s goodness-of-fit was adequate.

#### 3.4.2 Internal validation

**Figure 3:**
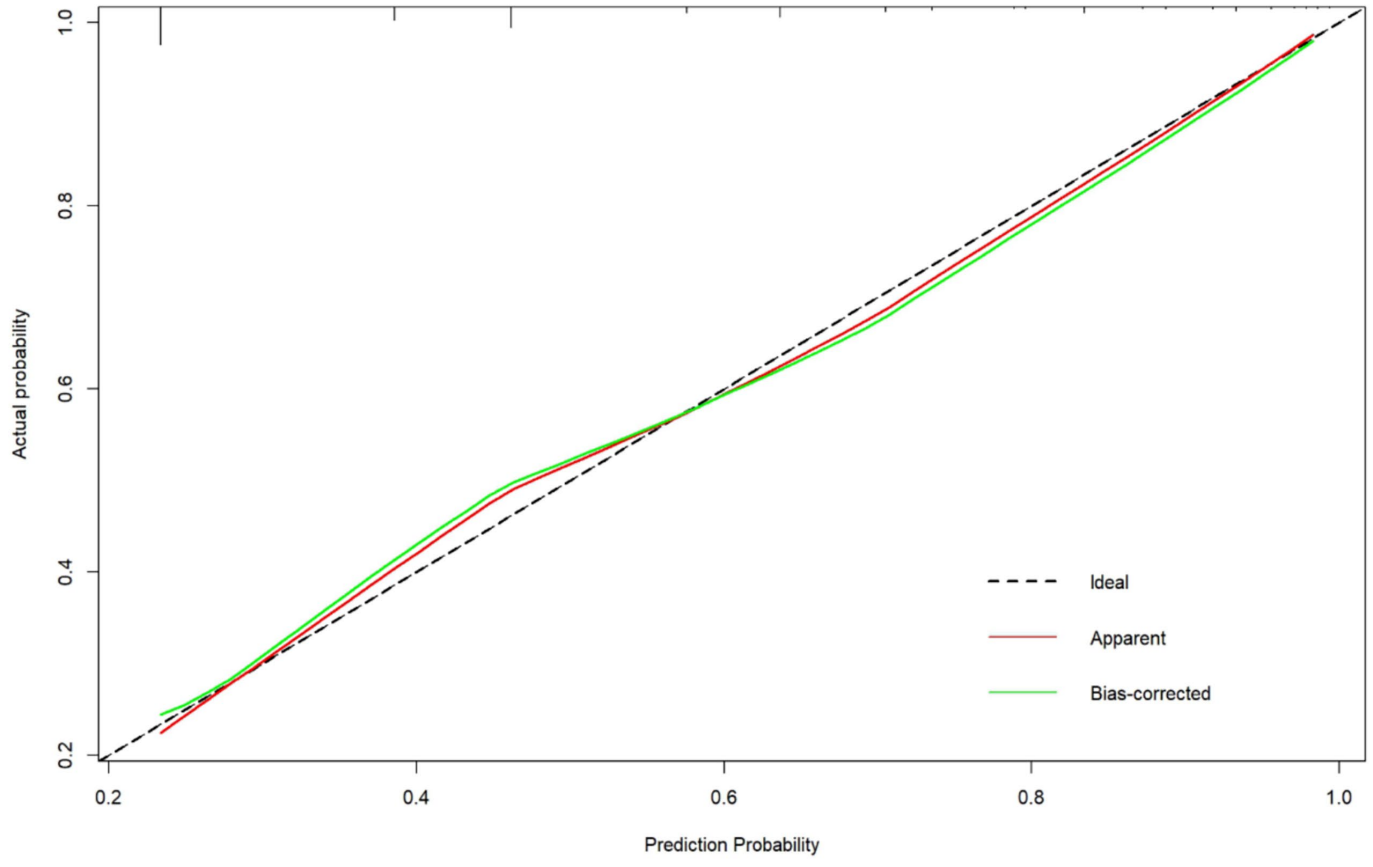
Calibration curve of the nomogram prediction model for forceps-assisted delivery. Internal validation was performed using 500 bootstrap resamples. The calibration curve, calibration intercept, calibration slope, and Brier score were used to assess calibration and overall prediction error. The bootstrap-corrected C-index was 0.771.

Internal validation was performed using 500 bootstrap resamples, yielding a corrected C-index of 0.771 and a mean absolute error of 0.04, suggesting acceptable internal performance in the derivation cohort. Calibration assessment revealed a Brier score of 0.190, a calibration intercept of 0.004, and a calibration slope of 0.991, all close to the ideal values. The calibration curve showed good agreement between predicted probabilities and actual risks (Figure 3).

#### 3.4.3 Clinical application value evaluation

**Figure 4:**
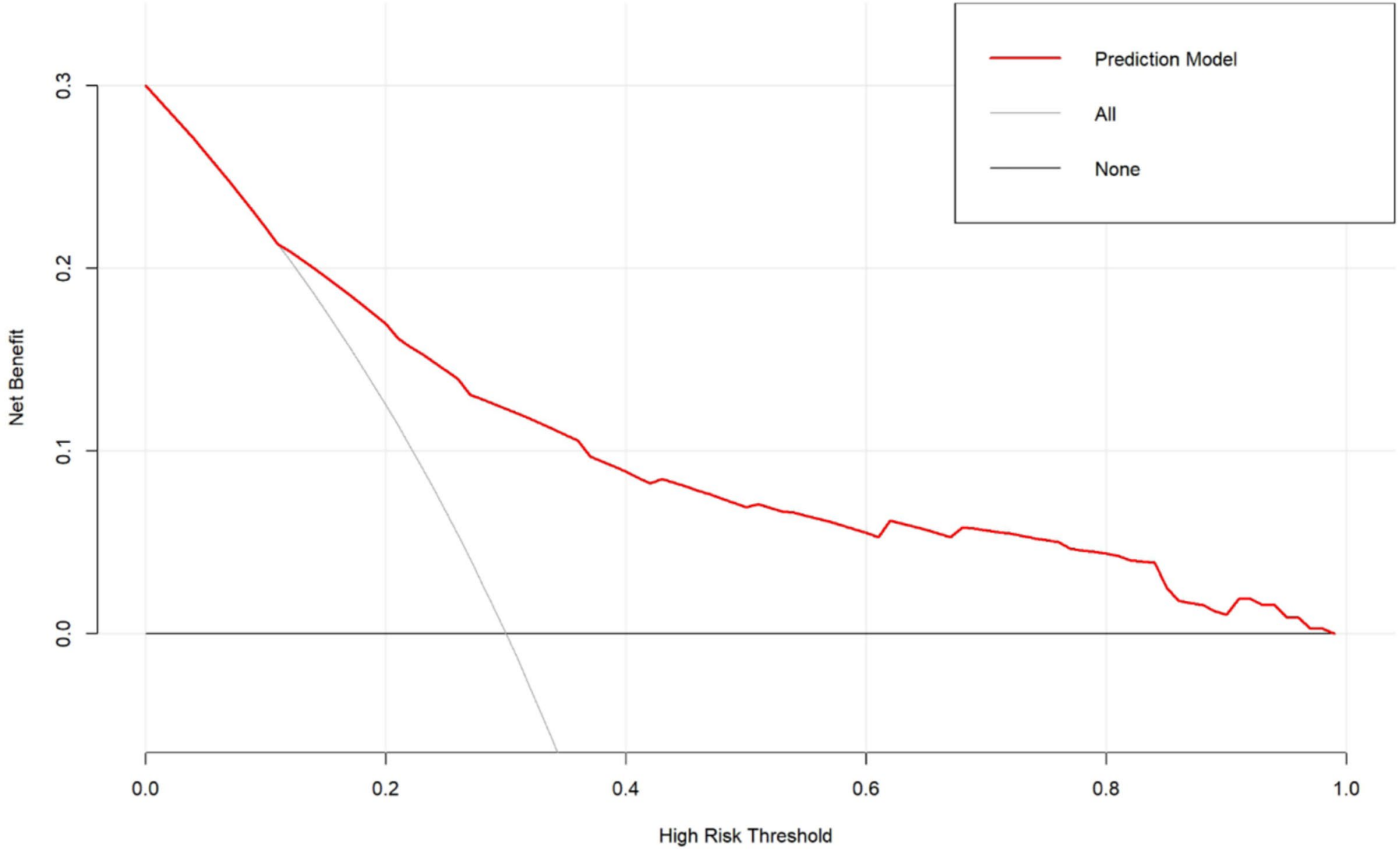
DCA of the prediction model for forceps-assisted delivery. The model showed potential net benefit within clinically plausible threshold probabilities, especially compared with the treat-all strategy, at thresholds up to 0.35.

The clinical utility of this predictive model was evaluated using DCA (Figure 4). The DCA suggested that the model provided a higher net benefit than the treat-all strategy within threshold probabilities up to 0.35, indicating potential clinical usefulness for early risk screening. At thresholds above 0.35, its net benefit did not exceed that of the no-intervention strategy, suggesting that the model may be more suitable for early risk screening in the early second stage of labor rather than for definitive high-risk decisions. Furthermore, because this study used a 1:1 matched design with an event rate of 50%, which is higher than the baseline level in the general obstetric population, the DCA results require validation or recalibration in real-world obstetric populations with lower baseline forceps-assisted delivery rates.

## 4 Discussion

Forceps-assisted delivery is a crucial intervention for resolving the second stage of labor arrest or fetal distress and avoiding emergency cesarean section. Appropriate use can expedite the second stage of labor and ensure maternal and infant safety; however, improper use significantly increases the risk of severe maternal and infant complications (13, 14). Through the analysis of 692 term singleton primipara, this study identified 5 independent risk factors for forceps-assisted delivery that can be assessed at the onset of the second stage of labor: pre-delivery BMI ≥ 28 kg/m^2^, fetal abdominal circumference ≥ 340 mm, MSF of grade II or III at the onset of the second stage of labor, non-OA fetal position at the onset of the second stage of labor, and active phase duration ≥ 120 min. All these indicators are derived from routine obstetric assessments, facilitating rapid bedside interpretation and timely risk stratification by clinicians, and are highly accessible and cost-effective in clinical practice. The predictive model constructed based on these factors shifts the risk assessment point to the onset of the second stage of labor, transforming the labor management model from passive response to proactive early warning. This provides an objective basis for the precise allocation of medical resources and improved delivery safety.

### 4.1 Independent risk factor analysis

Through multivariate analysis, this study ultimately identified five independent risk factors for forceps-assisted delivery that are obtainable at the onset of the second stage of labor. These findings are analyzed in conjunction with previous literature as follows.

First, this study included pre-delivery BMI in the analysis and found that a BMI ≥ 28 kg/m^2^ is an independent risk factor for forceps-assisted delivery. This finding is consistent with domestic and international research on obesity and dystocia (15, 16), which indicates that obesity and excessive gestational weight gain increase the risk of operative vaginal delivery and cesarean section. The potential mechanisms may include increased pelvic soft tissue in obese pregnant women, which affects the birth canal space and the descent of the fetal head. Concurrently, obesity is often associated with the risk of macrosomia (17). The synergistic effect of these two factors can easily prolong labor, maternal exhaustion, and fetal distress, thereby increasing the risk of forceps-assisted delivery (18). Given that this study could not obtain pre-pregnancy weight, using pre-delivery maternal BMI for assessment is more clinically feasible and also suggests the importance of strengthening gestational weight management.

Second, in terms of fetal indicators, the fetal abdominal circumference ≥ 340 mm was identified as an independent predictor of forceps-assisted delivery. A larger fetal abdominal circumference may reflect greater fetal trunk size and potential difficulty in fetal descent, thereby increasing the likelihood of operative vaginal delivery. Because fetal abdominal circumference is routinely available before or at the onset of the second stage of labor, it has practical value for early intrapartum risk stratification. Research indicates a strong link between fetal abdominal circumference and newborn birth weight (19, 20). During late pregnancy, fetal biparietal diameter and head circumference show relatively stable growth, but can be affected by factors such as fetal head descent and cranial bone overlapping, potentially underestimating the accuracy of fetal weight assessment. However, abdominal circumference is not affected by these factors, providing a more stable and accurate reflection of fetal trunk development and nutritional status. It is a critical adjunct for evaluating the head–pelvis relationship.

Third, OA is the most advantageous position for delivery. Fetal non-OA orientation is recognized as a significant cause of cephalopelvic disproportion and prolonged vaginal delivery. Our findings are consistent with the reports by scholars such as Foggin (21) and Zhao (22). Abnormal fetal positions (such as occiput transverse or occiput posterior positions) affect the mechanics of fetal head descent, increase labor resistance, prolong labor, and thus increase the likelihood of cesarean delivery or instrumental vaginal delivery. Consequently, accurate evaluation of fetal position via vaginal examination or ultrasound during labor, and prompt, targeted interventions (e.g., maternal position adjustments and manual cephalic rotation) for abnormal fetal positions, are vital for correcting these issues and facilitating natural childbirth.

Furthermore, this study also found that MSF of grade II or III at the onset of the second stage of labor is an independent risk factor for the use of forceps. Although MSF cannot be used as a specific diagnostic criterion for fetal hypoxia alone, its occurrence may be related to the duration of the first stage of labor (23). Longer labor durations are more likely to lead to abnormal fetal monitoring and fetal distress, thus prompting physicians to decide on instrumental delivery with forceps to expedite the end of labor. Therefore, the study focuses on the importance of the amniotic fluid status assessment during labor observation. Clinical decisions should be made through comprehensive analysis and careful judgment, integrating real-time fetal monitoring, labor progress, and other high-risk factors.

Finally, this study reports an independent correlation between active phase duration ≥ 120 min and the use of forceps. According to the monograph *Dystocia*, the active phase generally requires 1.5–2 h. In this study, the median active phase duration in the forceps-assisted delivery group was 110 min, making 120 min a clinically reasonable critical threshold. The observation of labor duration is crucial for dystocia, and the active phase is a key stage of labor progress. When there is a trend of a prolonged active phase, it indicates the need to re-evaluate fetal size, fetal presentation, pelvic conditions, and other factors to promptly identify the causes of dystocia and implement effective interventions. Currently, there are few reports on the relationship between active phase duration and forceps-assisted delivery; this study provides a reference.

### 4.2 Advantages and applications of the model

The primary advantage of this study is its potential for early risk stratification. The predictive model constructed incorporates five independent risk factors accessible at the onset of the second stage of labor, providing an objective and quantitative reference tool to identify higher-risk women in a timely manner. Notably, the influence of MSF and active phase duration at the onset of the second stage of labor on the likelihood of forceps-assisted delivery has received insufficient attention in earlier research, highlighting the novel contribution of this model.

It is crucial to emphasize that while a single risk factor has limited predictive value, the synergistic effect of multiple factors significantly increases the risk of dystocia. Therefore, the model’s value lies in its comprehensive assessment of the combined effects of multiple factors. In clinical application, the model may support early recognition of women at higher risk, enabling clinicians to prepare and mobilize resources, such as consultation with experienced obstetricians and ensuring the availability of neonatal support, rather than serving as a stand-alone decision-making tool for forceps-assisted delivery. In clinical practice, the model may support early recognition of women at relatively high risk, allowing clinicians to prepare and mobilize resources, such as senior obstetrician review, an experienced forceps-assisted delivery team, intensified intrapartum monitoring, and neonatal support. However, given its moderate sensitivity, the model should not be used as a stand-alone decision-making tool for determining whether forceps-assisted delivery should be performed.

The DCA curve suggested that the model may provide a potential net benefit within low-to-moderate clinically plausible threshold probabilities up to 0.35, corresponding to early second-stage labor scenarios in which maternal and fetal risks remain uncertain, and clinicians may need to prepare obstetric and neonatal resources in advance based on preliminary risk stratification. In the high-risk threshold range, the model shows no significant advantage, which aligns with the clinical reality that clear indications for forceps delivery at this stage render model-dependent decision-making unnecessary. The core role of this model is as an early risk warning and stratification tool in the early second stage of labor, integrating dispersed risk factors into an overall risk probability. This helps shift intrapartum management from a reactive approach to a proactive early warning, providing actionable evidence for optimizing resource allocation and ensuring maternal and neonatal safety.

In future implementation settings, the model may support early recognition of women at relatively higher risk and assist clinicians in preparing intrapartum resources. Potential actions include senior obstetrician review, intensified fetal monitoring, preparation of a qualified forceps-assisted delivery team, and neonatal team readiness. However, these actions should be regarded as potential use cases for future implementation studies rather than clinical benefits directly demonstrated by the current retrospective design.

### 4.3 Limitations and prospects of the study

This study has several limitations. First, clinical practice typically relies on external pelvic measurements or pelvic outlet measurements, resulting in a lack of standardized and quantified data for more detailed pelvic measurements, such as the diagonal diameter or ischial spine protrusion. Pelvic conditions, although critical anatomical factors influencing fetal head descent and mode of delivery, were not directly included as predictors to ensure the mode’s accessibility, stability, and reproducibility. Although we effectively compensated for this by incorporating variables related to cephalopelvic disproportion (CPD) through experienced physicians’ clinical pelvic examinations, combined with fetal weight, fetal position, station, and labor progression, the model’s predictive power remains insufficient for pregnant women with potential pelvic abnormalities. To prioritize bedside clinical operability and facilitate rapid risk assessment during labor, continuous variables were dichotomized in the present model. Although this approach improves clinical interpretability and ease of use, it may have resulted in some loss of prognostic information. Therefore, future studies should compare the current dichotomized model with models incorporating continuous predictors to further evaluate the robustness and clinical applicability of this modeling strategy.

Second, in terms of research design, the 1:1 neighboring case matching design balanced time-related confounding factors, which artificially increased the event occurrence rate. Thus, external validation and calibration were based on the actual clinical incidence rate. This is a single-center, retrospective study with a relatively small sample size, which may lead to statistical bias in the results. Furthermore, only internal validation was performed, which may result in statistical bias and limit the model’s generalizability. Finally, factors such as pre-pregnancy weight, gestational weight gain, perineum conditions, and psychological state during labor were not included in the analysis due to data accessibility issues.

To address these limitations, future studies should prioritize prospective multicenter external validation, protocol-aware model updating and recalibration, and the incorporation of standardized pelvic imaging and maternal psychosocial measures; in parallel, integrating the model into an artificial intelligence-enabled intrapartum monitoring framework and evaluating its real-world impact through implementation studies will be essential to translate risk prediction into reliable, actionable decision support across diverse clinical settings.

## 5 Conclusion

This model enables early risk stratification for forceps delivery in term singleton primiparas using routine clinical variables and demonstrates acceptable internal validation, although further external validation is needed to establish its clinical utility.

## Data Availability

The original contributions presented in the study are included in the article/supplementary material, further inquiries can be directed to the corresponding author/s.
